# Linking neuroanatomical abnormalities in autism spectrum disorder with gene expression of candidate ASD genes: A meta-analytic and network-oriented approach

**DOI:** 10.1371/journal.pone.0277466

**Published:** 2022-11-28

**Authors:** Alessia Camasio, Elisa Panzeri, Lorenzo Mancuso, Tommaso Costa, Jordi Manuello, Mario Ferraro, Sergio Duca, Franco Cauda, Donato Liloia

**Affiliations:** 1 GCS-fMRI, Koelliker Hospital, Turin, Italy; 2 Department of Physics, University of Turin, Turin, Italy; 3 School of Biological Sciences, University of Leicester, Leicester, United Kingdom; 4 Focus Lab, Department of Psychology, University of Turin, Turin, Italy; Museo Storico della Fisica e Centro Studi e Ricerche Enrico Fermi, ITALY

## Abstract

**Background:**

Autism spectrum disorder (ASD) is a set of developmental conditions with widespread neuroanatomical abnormalities and a strong genetic basis. Although neuroimaging studies have indicated anatomical changes in grey matter (GM) morphometry, their associations with gene expression remain elusive.

**Methods:**

Here, we aim to understand how gene expression correlates with neuroanatomical atypicalities in ASD. To do so, we performed a coordinate-based meta-analysis to determine the common GM variation pattern in the autistic brain. From the Allen Human Brain Atlas, we selected eight genes from the SHANK, NRXN, NLGN family and MECP2, which have been implicated with ASD, particularly in regards to altered synaptic transmission and plasticity. The gene expression maps for each gene were built. We then assessed the correlation between the gene expression maps and the GM alteration maps. Lastly, we projected the obtained clusters of GM alteration-gene correlations on top of the canonical resting state networks, in order to provide a functional characterization of the structural evidence.

**Results:**

We found that gene expression of most genes correlated with GM alteration (both increase and decrease) in regions located in the default mode network. Decreased GM was also correlated with gene expression of some ASD genes in areas associated with the dorsal attention and cerebellar network. Lastly, single genes were found to be significantly correlated with increased GM in areas located in the somatomotor, limbic and ganglia/thalamus networks.

**Conclusions:**

This approach allowed us to combine the well beaten path of genetic and brain imaging in a novel way, to specifically investigate the relation between gene expression and brain with structural damage, and individuate genes of potential interest for further investigation in the functional domain.

## Introduction

Autism spectrum disorder (ASD) is a cluster of neurodevelopmental conditions characterized by impairments in socio-communicative ability, repetitive behaviors, and abnormal sensory perception [1]. The etiology of ASD is multifactorial and not comprehensively understood, with both genetic and environmental factors playing a role [2, 3].

In the last decade, research in the genetic component of ASD has been fruitful, with hundreds of genes having been identified as associated with the disorder [4]. Moreover, anomalies at the level of the synapse have been identified since long ago as one of the main underlying mechanisms of ASD [5, 6], as well as related to abnormal brain connectivity between neuronal subpopulations and systems [7]. As individuated in a review by Guang and colleagues [5], as well as in a transcriptomic analysis by He and colleagues [6], SHANK3, SHANK1, SHANK2, NLGN3, NGLN4X, MECP2, and CNTNAP2 genes are of particular interest for ASD research due to their role in synaptic formation, maintenance, and transmission. Specifically, the SHANK family encodes for essential scaffold proteins in the postsynaptic density of excitatory synapses [8], with mutations leading to altered levels of postsynaptic density proteins, synapse morphology, and excitatory transmission [8–11]. NLGN3 and NLGN4X encode for postsynaptic cell adhesion proteins, with NLGN3’s mutations having been reported to increase inhibitory transmission and NLGN4X to cause altered excitatory transmission [12–14]. Moreover, defects in NLGN3 and NLGN4X have been reported to lead to impaired synaptogenesis [12]. Presynaptic cell adhesion protein NRXN1’s role in synaptic disruption has been attributed to altered Ca^2+^ entry at the synapse thus impairing neurotransmitter release [5]. Dysfunction in CNTNAP2 has been linked to impaired axonal growth and reduced dendritic arborisation of inhibitory interneurons as well as impaired synaptic transmission [15, 16]. MECP2 encodes for a regulator of chromatin remodeling mostly responsible for silencing gene expression, with deficiencies in the gene expression having been reported to induce decreased excitatory transmission due to a decrease in synapse plasticity and number [17, 18].

The autistic brain has been studied extensively with various approaches, from cellular resolution to whole-brain imaging. One such technique is voxel-based morphometry (VBM) [19], which allows to quantify regional neuroanatomical differences in volume/concentration in individuals with ASD compared to typically developing controls (TDCs). Moreover, using coordinate-based meta-analytic (CBMA) approaches, different authors have identified spatially consistent gray matter (GM) abnormalities across VBM published findings regarding several ASD groups analyzed worldwide. To note, these abnormal territories tend to encompass a wide set of multimodal, perceptual, subcortical, and cerebellar areas [20–24]. These important findings notwithstanding, the pathophysiological mechanisms and genetic effects that underpin atypical GM patterns in ASD remain largely unappreciated. A more comprehensive insight into the disease may be given by combining the genetic and neuroimaging data. In recent years, studies linking genomic variation to neuroimaging meta-analysis are starting to appear. For example, Grasby and colleagues [25] combined genetic and magnetic resonance imaging data to link genetic variation to cortical surface area and thickness. The authors demonstrated that genetic variants associated with brain morphology are also associated with cognitive function as well as neuropsychiatric diseases [25]. Also, Lau and colleagues [26] related gradients of gene expression to brain structure and development.

Resting-state functional magnetic resonance imaging (rs-fMRI) [27], a technique able to estimate the intrinsic activity interactions between brain regions that occur during the rest (i.e. when no active task is being performed and the brain is not actively engaged), represents another useful tool to further our understanding of brain disorders [28–32]. A number of resting-state networks (RSNs) have been identified via rs-fMRI [27]. As suggested by different studies [33–36], these RSNs reveal patterns of activity that are consistent across subjects and reproducible across participants and time. In particular, canonical RSNs such as the default mode network (DMN), salience network (SN), dorsal attention network (DAN), and sensorimotor network (SMN) were often found to be functionally or anatomically altered in ASD (for a review on the topic see [37]). Moreover, RSNs are highly inheritable, and influenced by genomic factors [38, 39]. Therefore, RSNs could be a useful perspective to interpret the genetic spatial variation associated with ASD morphological alteration.

In this study, we aim to investigate the relation between brain anatomical alterations and genetic expression of the following genes: SHANK3, SHANK1, SHANK2, NLGN3, NLGN4X, MECP2 and CNTNAP2. These genes are selected because of their relation to ASD pathology and role in synaptic transmission and plasticity [5, 6]. Specifically, we aim to identify the most consistent brain anatomical alterations in ASD and link these alterations with the corresponding genetic expression. Moreover, we also complement the gene-structure results with a functional interpretation informed by the canonical resting state networks. This choice is motived by an increasing number of experimental efforts suggesting that the development of neuroanatomical alteration patterns in brain disorders are influenced by functional connectivity constraints [29–31, 40, 41]. Therefore, we expect to see the neuronal alteration and the selected genes distributed according to spatial patterns meaningful for brain function and functional connectivity [29]. What is more, we expect that, when assigning those altered regions to canonical RSNs, high-order networks typically associated with ASD, such as the DMN, would be found to be particularly related to our set of genes [42].

## Materials and methods

### Gene expression data

The genetic expression data were obtained from the Allen Human Brain Atlas (AHBA; https://human.brain-map.org/) [43, 44]. This resource stores anatomical and histological data (including the RNA microarray data used in this study) collected from six healthy human specimens with no known neurological disease history (one female; age range = 24–57 years; mean age = 42.5 years). Two specimens contain data from the entire brain, whereas the remaining four include data from the left hemisphere only. The microarray analysis was originally performed as follows: the brains tissues were partitioned into smaller blocks according to their anatomical roles (i.e. if they were cortical or subcortical structures). A minimum amount of tissue was collected from each block and subsequently processed for mRNA isolation. Microarray analysis was carried out by a third-company party (Beckman Coulter Genomics). The data were then normalized to be included in the Atlas [45].

Microarray data were extracted from the AHBA using the Allen Software Development Kit [43]. We thus obtained gene expression data for specific brain locations that we labeled with Talairach coordinates for each of the six specimens. For each gene focus we created Voronoi polygons having the gene expression point as the barycenter using the Voronoi tessellation algorithm [46], as done by Torta and colleagues [47]. The Voronoi tessellation is a decomposition of metric space by distances between sets of points. We assigned to each Voronoi polygon the same gene expression value as the barycenter. We thus obtained six brain maps for a single gene—one for each specimen of the AHBA—that we averaged to obtain a single voxel-wise brain map for each gene. In case of specimens having data only in the left hemisphere, we supposed a symmetry of the data between the two hemispheres, obtaining gene expression for the whole-brain [29]. For the whole-brain gene expression maps see also S1 Fig.

### Voxel-based morphometry data

The VBM data were retrieved from the BrainMap and Medline databases. The BrainMap database is part of the BrainMap Project and currently contains a collection of more than 3000 peer-reviewed VBM experiments [48–51]. Medline is an online database developed by the U.S. National Library of Medicine, containing more that 26 million published works in the field of biomedicine. The data from BrainMap were accessed through the Sleuth 2.4 software package (http://www.brainmap.org/sleuth/). Two standardized search algorithms were employed as described below to individuate experiments reporting either foci of GM decrease (i.e. neuroanatomical hypotrophy; ASD < TDCs) or increase (i.e. neuroanatomical hypertrophy; ASD > TDCs) in subjects with ASD compared to TDCs:

GM decrease query: *[Experiments Contrast is Gray Matter] + [Experiments Context is Disease Effects] + [Subjects Diagnosis is Autism Spectrum Disorders] + [Experiments Observed Changes is Controls > Patients]*;
GM increase query: *[Experiments Contrast is Gray Matter] + [Experiments Context is Disease Effects] + [Subjects Diagnosis is Autism Spectrum Disorders] + [Experiments Observed Changes is Controls < Patients]*.

To retrieve eventual articles not stored in the BrainMap database, we also conducted a systematic search in the Medline database using the PubMed search engine as described below:

*(“Autism Spectrum Disorders” [Title/Abstract] OR “ASD” [Title/Abstract] OR “Autism”) AND (“Voxel-Based Morphometry” [Title/Abstract] OR “VBM” [Title/Abstract])*.

The search design adhered to the PRISMA Statement international guidelines [52, 53] (see also the checklist in the S1 Table). This study was also compliant with the latest guidelines for the implementation of neuroimaging CBMA [54]. We selected peer-reviewed articles containing experiments conducted using whole-brain VBM analysis, for which the results were reported either in Talairach (TAL) or Montreal Neurological Institute (MNI) stereotaxic space (i.e. x-y-z coordinates). Moreover, the experiments must have reported alteration of GM in subjects with ASD compared between-group to TDCs. We discarded all the experiments which were based either on samples of less than 10 participants, or on a region-of-interest (ROI) analysis [54–56]. Moreover, we minimized potential bias regarding the analysis of overlapping ASD populations both within and between published data. In details, in the case of multiple VBM experiments included in a single article we included only the x-y-z coordinates coming from those experiments analyzing no redundant subjects with ASD (see also S2 Table). In the case of two or more articles published by the same first author, we evaluated the presence of the duplicated clinical population and results, including eventually only the first published paper.

### Resting-state functional networks atlas

To interpret the correlation between the gene expression and the GM alteration maps in a large-scale networking perspective (details below), the Yeo’s atlas [57] was adopted. Using resting-state functional magnetic resonance imaging (rs-fMRI) data from 1,000 typically developing subjects, Yeo and colleagues [56] parcellated the human cerebral cortex in 7 large-scale functional networks, namely the default mode network (DMN), dorsal attention network (DAN), somatomotor network (SMN), frontoparietal network (FPN), limbic network, visual network, and ventral attention/salience network (VAN/SN).

However, since previous CBMAs on ASD also found consistent neuroanatomical abnormalities at the level of cerebellum and subcortical nuclei [20–24], two other networks representing the basal ganglia/thalamus (BG/Thal) and the cerebellum were included in the analysis. Specifically, the BG/Thal and cerebellar volumes were obtained from the Brainnetome Atlas [58].

### Anatomical likelihood estimation meta-analysis

To evaluate consistent patterns of GM alteration across the selected VBM experiments, we used the GingerALE software package (v. 3.0.2; https://www.brainmap.org/ale/), employing the current version of the anatomical likelihood estimation (ALE) algorithm [59–62]. ALE is a quantitative coordinate-based meta-analytical method that allows to estimate consistent areas of neuroanatomical variation across independent experiments. In the ALE analysis, a three-dimensional Gaussian probability distribution is constructed around every focus of each experiment. Let *X*_*i*_ denote the *i*^th^ focus is in a given voxel. The probability the *X*_*i*_ is located at voxel x-y-z is

$$p(X_{i})=\frac{1}{\sigma^{3}\sqrt{(2\pi {)}^{3}}}e^{-{d_{i}}^{2}/2\sigma^{2}}$$

Where *d*_*i*_ is the Euclidean distance between the voxel and the given focus and the standard deviation of the gaussian distribution is determined through the full-width half-maximum (FWHM) as:

$$\sigma =\frac{FWHM}{\sqrt{8ln2}}$$

A final ALE map was obtained from the union of all the maps calculated from each experiment [62]. The level of significance was based on a null distribution. A threshold P-value was derived by a non-parametric permutation test. In this test, *n* random foci are generated, where *n* equals the number of foci in the ALE meta-analysis, and the corresponding ALE values for these random foci are computed. The set of ALE values calculated form the null distribution of the statistic. The GingerALE software generates various output images: the thresholded map with the Monte Carlo procedure, the unthresholded map, the Z map in which the ALE map is converted in Z-points, and the map of the significant clusters based on the results of the ALE and the dimension of the clusters, respectively. To note, the cluster map is a logical map in which each cluster is labeled with a different color and number. This logic map is useful for later selecting the clusters to analyze.

We carried out two separate ALE analyses to determine the most spatially consistent patterns of GM decrease (i.e. hypotrophy; ASD < TDCs) and increase (i.e. hypertrophy; ASD > TDCs), respectively, across selected experiments. Since the ALE maps served as input to a set of network-oriented correlational analyses with their own statistical significance testing (details in the next section), we opted for a liberal threshold at this step in order to not miss any potential areas of converging GM alteration. Therefore, the significance threshold was set at P-value < 0.05 uncorrected and minimum cluster size = 150 mm^3^. All coordinates of the experiments taken in consideration were placed in the Talairach space. Original MNI coordinates were transformed in Talairach space via the icbm2tal algorithm [63].

### Correlation between ALE and gene expression maps

We then investigated the relation between GM alteration assessed through the ALE and gene expression. This was iteratively performed for each cluster of GM alteration, distinguishing decrease and increase, and for each gene expression map (i.e., cluster1:decrease-SHANK3, cluster1:increase-SHANK3, cluster1:decrease-SHANK1, etc.). For each cluster in a given alteration-gene expression couple, we calculated the Pearson’s correlation between the ALE values (converted in z-points) of the voxels inside the cluster and the values of gene expression (again, in z-points) of the same voxels, applying the Fisher’s r to t transformation. The resulting *r* value was assigned to all the voxels of that cluster. We thus obtained 16 gene-specific maps, 8 related with GM decrease and 8 related with GM increase.

Next, we wanted to assess if such cluster-wise association varied between RSNs. To do so, we used the Yeo’s cortical atlas [57] (complemented with regions representing the basal ganglia, the thalamus and the cerebellum as explained above [58]), converted in Talairach space using FLIRT [64, 65]. Each of the previously obtained 16 correlation maps was then projected onto the RSNs atlas. The correlation coefficients (if different from 0) of the voxels inside each network were averaged to obtain the network-level mean gene-alteration association. To assess statistical significance, we used a Monte Carlo method to evaluate if a RSN gene-alteration association was higher than chance. We randomly permuted the correlation clusters (N = 10,000), reassigning the value of correlation of each cluster to another one. Then, for each iteration, we recalculated each network’s mean correlation value, thus obtaining a distribution of mean correlations for every RSN. We then calculated the significance threshold with a 95% confidence interval (CI). Genes correlation values falling above the 95% CI were considered to be statistically significant.

## Results

### Descriptive overview

Of the initial 517 potential published articles, only 51 fit the inclusion criteria. They included a total of 80 VBM experiments reporting 541 coordinates of GM alterations, subdivided in 244 decreases and 297 increases, respectively (S2 Fig). A total of 4849 subjects were included, for a total of 2366 subjects with ASD (372 female; mean age (group range) = 18.3 years (4.4–37.9) and 2483 TDCs (430 female; mean age (group range) = 17.8 years (4.4.–39.0). Demographic, methodological, and diagnostic details of the VBM data are shown in S2–S4 Tables, respectively.

### Correlation of decrease clusters

**Fig 1:**
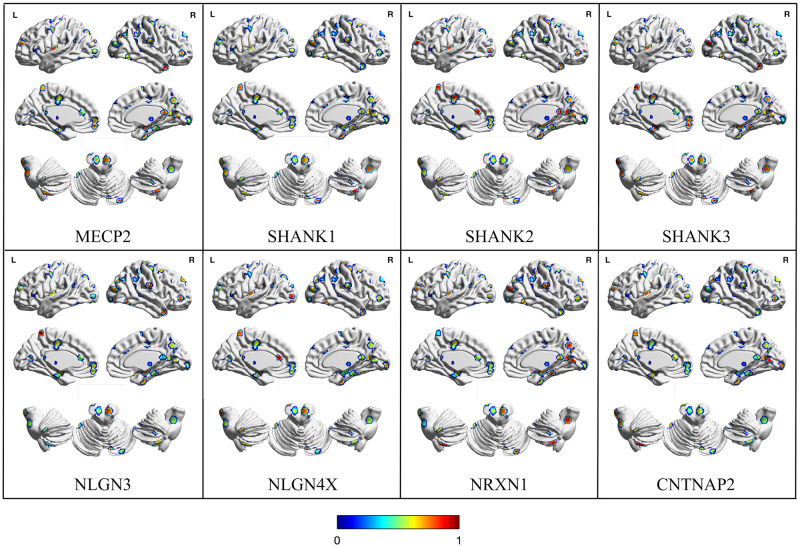
Map with the correlation between gene expression and ALE-derived gray matter decrease of each cluster. SMN: Sensorimotor Network, DAN: Dorsal Attention Network, VAN/SN: Ventral Attention/Salience Network, DMN: Default Mode Network, BG/Thal: Basal Ganglia/Thalamus.

Fig 1 (obtained with BrainNet Viewer [66]) represents a graphical overview of the correlation value between gene expression and ALE value for each cluster of GM decrease. GM decrease clusters are located in distributed parts of the brain consistently found by previous CBMAs [20, 24, 67, 68], including the left posterior insula, around left and right central sulcus, along the cingulate cortex, in the bilateral medial temporal cortex, in the precuneus and across the lateral parietal and occipital cortices. The cerebellum shows several clusters of GM decrease as well.

**Fig 2:**
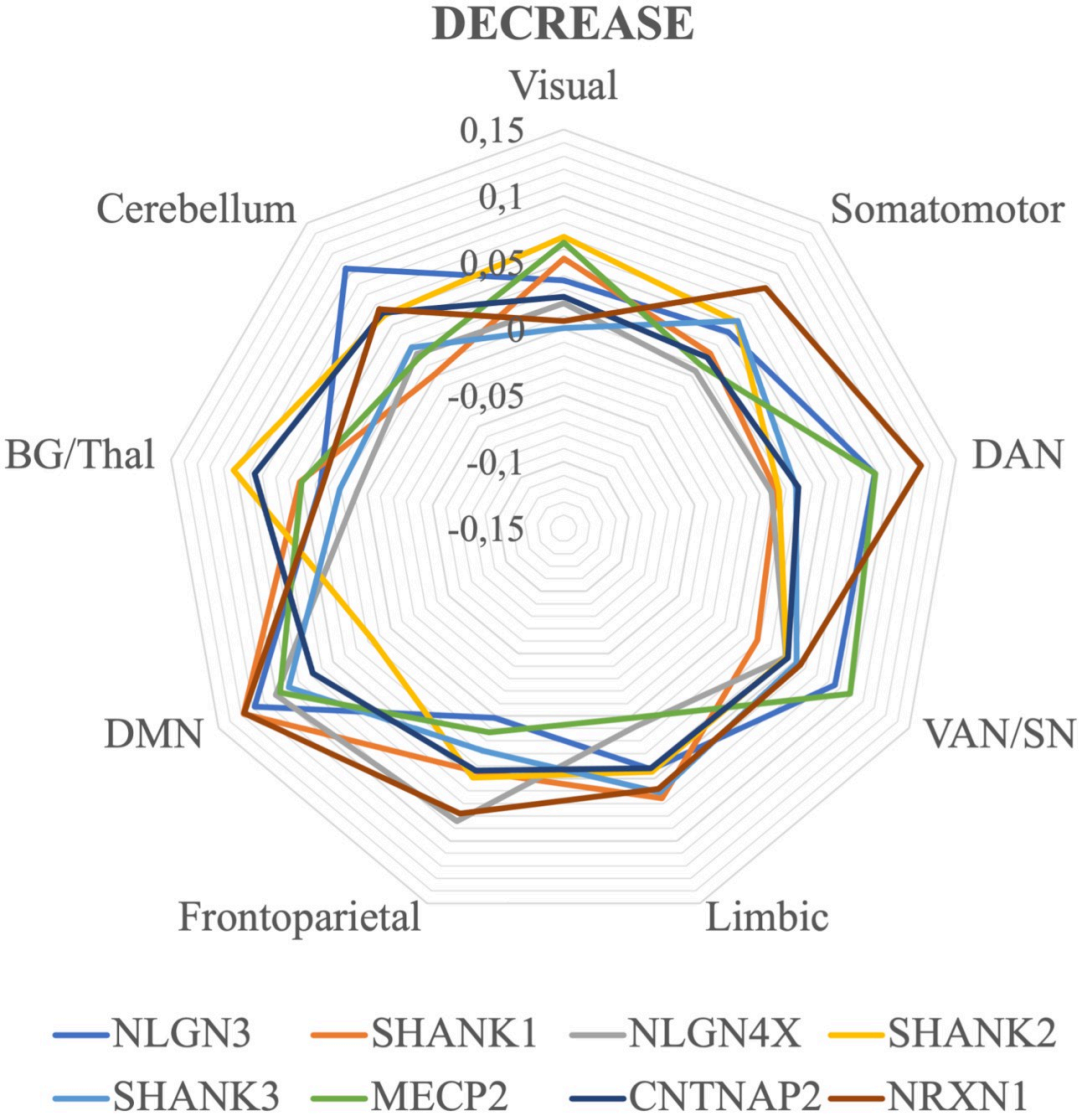
Average correlation between genetic expression and gray matter decreases values for each gene and functional network. The maps are visualized as three-dimensional cortical and cerebellar surfaces. Brain templates are in neurological convention (i.e. R is right, L is left). Color maps represent the correlation values for each gene expression selected. Both significant and non-significant values are represented. SMN: Sensorimotor Network, DAN: Dorsal Attention Network, VAN/SN: Ventral Attention/Salience Network, DMN: Default Mode Network, BG/Thal: Basal Ganglia/Thalamus.

Fig 2 illustrates the average correlation between gene expression and decrease ALE value for each gene and RSN. The correlation values and significance of each gene for each RSN are reported in Table 1. Although most genes are expressed homogeneously across networks, the DMN and the DAN make significant exceptions. In the DMN, the genes NLGN4X, NRXN1, NLGN3, SHANK1, SHANK3, MECP2 and CNTNAP2 seem to be significantly more correlated than chance (p = 0.05; 10,000 permutation runs). Similarly, NRXN1, SHANK3 and MECP2 have correlation values significantly higher than the null model in the DAN. Also, the cerebellum has one significantly correlated gene (i.e. NRXN1, r = 0.07).

**Table 1:** The correlation value of gene expression related to areas of GM decrease (ASD < TDCs) in Yeo’s [57] and Brainnetome’s [58] resting-state functional networks.

	MECP2	SHANK1	SHANK2	SHANK3	NLGN3	NLGN4X	NRXN1	CNTNAP2
Visual	0.07	0.05	0.07	0.01	0.04	0.02	0.01	0.02
SMN	0.01	0.02	0.05	0.05	0.04	0.01	0.09	0.02
DAN	***0*.*09***	0.01	0.01	0.03	***0*.*09***	0.01	***0*.*12***	0.03
VAN/SN	0.10	0.02	0.04	0.05	0.09	0.04	0.06	0.05
Limbic	0.01	0.07	0.05	0.06	0.04	0.01	0.06	0.04
FPN	0.01	0.04	0.05	0.03	0.01	0.08	0.08	0.04
DMN	***0*.*10***	***0*.*13***	0.02	***0*.*09***	***0*.*12***	***0*.*10***	***0*.*13***	0.07
BG/Thal	0.05	0.05	0.10	0.02	0.04	0.01	0.03	0.09
Cerebellum	0.02	0.01	0.06	0.03	0.11	0.02	***0*.*07***	0.06

The numbers in bold indicate significant values at p = 0.05 and 10,000 permutation runs. SMN: Sensorimotor Network, DAN: Dorsal Attention Network, VAN/SN: Ventral Attention/Salience Network, DMN: Default Mode Network, BG/Thal: Basal Ganglia/Thalamus.

### Correlation of increase clusters

**Fig 3:**
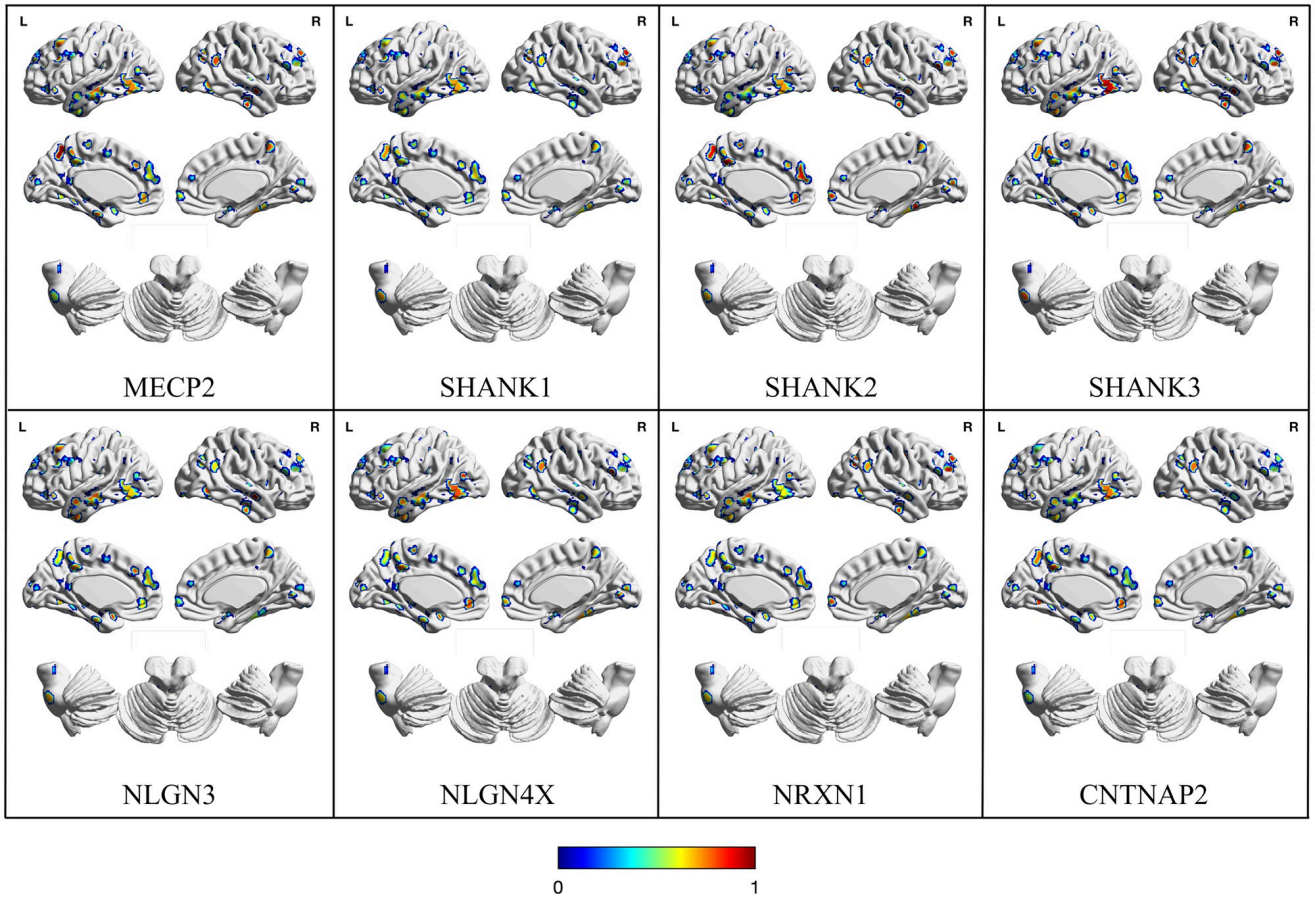
Map with the correlation between gene expression and ALE-derived gray matter increase of each cluster. SMN: Sensorimotor Network, DAN: Dorsal Attention Network, VAN/SN: Ventral Attention/Salience Network, DMN: Default Mode Network, BG/Thal: Basal Ganglia/Thalamus.

**Fig 4:**
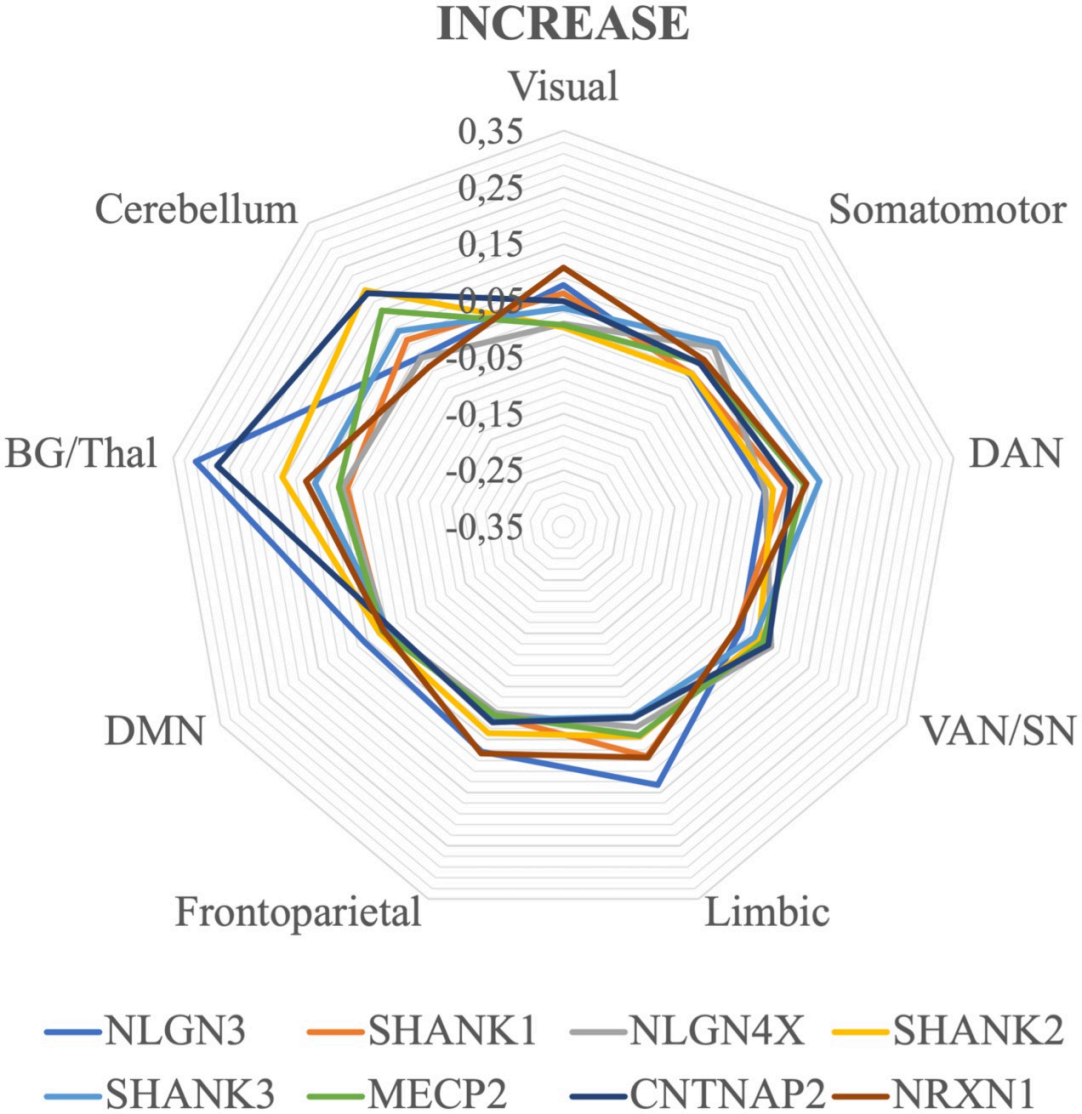
Average correlation between genetic expression and gray matter increases values for each gene and functional network. The maps are visualized as three-dimensional cortical and cerebellar surfaces. Brain templates are in neurological convention (i.e. R is right, L is left). Color maps represent the correlation values for each gene expression selected. Both significant and non-significant values are represented. SMN: Sensorimotor Network, DAN: Dorsal Attention Network, VAN/SN: Ventral Attention/Salience Network, DMN: Default Mode Network, BG/Thal: Basal Ganglia/Thalamus.

The correlation values between the gene expression and the GM increase ALE values are represented in Fig 3, while the average correlations of each network for each gene are shown in Fig 4. Here, the lateral prefrontal and temporal cortices are more involved than in the decrease maps, while the parietal and occipital lobes are relatively preserved. Compared to the GM decrease maps, the cerebellum seems to be less altered as well. Curiously, the midline cortical structures of the left hemisphere show several clusters of increases, while their homotopic counterparts display less alterations of the same kind. Apart from these observations, the various genes show different local correlations, with no obvious pattern to report.

As can be seen in the radar plot in Fig 4, most genes correlate similarly with the increase ALE values of the various RSNs, with the exception of the BG/THAL and the cerebellum. However, such a visible trend is not confirmed by the Monte Carlo procedure. The significance of the correlation values is represented in Table 2. Compared to the decreases results, there are less significant values per RSNs. The cerebellum is not significantly more correlated to any gene than chance. The discrepancy between the Monte Carlo test and the average cerebellar values observed in the radar graph is likely due to the fact that such structure is characterized by only two increase clusters. The networks revealing at least an average correlation between gene expression and GM increases are the SMN (i.e. NLGN4X, r = 0.06), the limbic (i.e. NLGN3, r = 0.14), the DMN (i.e. NLGN3, r = 0.06) and BG/Thal (i.e. NLGN3, r = 0.31; CNTNAP2, r = 0.27). To note, NLGN3 is the only gene significantly correlated with increases in more than one network, and BG/Thal is the only system presenting more than one significant correlation. In any case, it might be worth noting that the DMN is the only functional system showing significant values in both states of GM alteration (i.e. decrease and increase), highlighting a potentially significant role of the network in the pathological structural alterations found in ASD.

**Table 2:** The correlation value of gene expression related to areas of GM increase (ASD > TDCs) in Yeo’s [57] and Brainnetome’s [58] resting-state functional networks.

	MECP2	SHANK1	SHANK2	SHANK3	NLGN3	NLGN4X	NRXN1	CNTNAP2
Visual	0.01	0.06	0.01	0.04	0.08	0.01	0.11	0.05
SMN	0.03	0.01	0.01	0.07	0.01	***0*.*06***	0.04	0.03
DAN	0.08	0.05	0.03	0.11	0.01	0.01	0.09	0.06
VAN/SN	0.06	0.01	0.05	0.04	0.01	0.07	0.01	0.07
Limbic	0.04	0.08	0.05	0.01	***0*.*14***	0.03	0.08	0.01
FPN	0.01	0.01	0.04	0.01	0.07	0.01	0.08	0.02
DMN	0.02	0.02	0.03	0.01	***0*.*06***	0.01	0.02	0.01
BG/Thal	0.05	0.04	0.15	0.10	***0*.*31***	0.05	0.11	***0*.*27***
Cerebellum	0.15	0.08	0.20	0.10	0.04	0.04	0.02	0.19

The numbers in bold indicate significant values at p = 0.05 and 10,000 permutation runs. SMN: Sensorimotor Network, DAN: Dorsal Attention Network, VAN/SN: Ventral Attention/Salience Network, DMN: Default Mode Network, BG/Thal: Basal Ganglia/Thalamus.

## Discussion

In this study we investigated the relation between genetic and neuroanatomical data in the autistic brain. Adopting a VBM meta-analytic and functional resting-state network perspective, we correlated consistent patterns of brain variation with eight gene expressions previously related with both synaptopathology and ASD.

We found that the DMN-related alteration was significantly correlated with gene expression of at least one candidate ASD gene in both decrease and increase neuroanatomical analyses. Specifically, in relation to GM decrease results, the DMN pattern correlates significantly with six out of eight of the genes selected. The DMN is a resting-state brain network extensively associated with psychological functions deficient in the disorder, including mentalizing, empathy, episodic memory, and Theory of Mind [41, 69, 70]. Moreover, dysfunction in its connectivity has been linked with ASD [41, 70, 71]. In light of this, our results confirm and extend previous literature, highlighting the DMN as a network of particular interest in the pathology of ASD. Moreover, we identified MECP2, SHANK1, SHANK3, NLGN3, NLGN4X and NRXN1 as significantly expressed, therefore opening up a path of investigation into how altering their function and expression in specific areas that are functionally connected in the DMN may be reflected in pathogenic anatomical changes.

The DAN-related alteration results significantly correlated with three genes in the VBM decrease but not increase data. The DAN is another RSN of particular interest in the pathology in ASD due to its association to attentional functions, one of the most consistent domain deficiencies across the spectrum [72]. Our results supplement a study by Farrant and Uddin [73], revealing that individuals with ASD display lower functional connectivity between nodes of the DAN compared to TDCs. Interestingly, we found significance for such networks only in the decrease analysis, highlighting a possible correspondence between functional hypo-connectivity, genetic expression, and GM morphometrical reductions. We also found that in the VBM decrease data there was a significant correlation between the cerebellar alteration pattern and the NRXN1. This result is in line with previous MRI research that shows a volumetric reduction of Purkinje cells, as well as a marked hypo-connectivity between the cerebellum and the cortical regions, in both pediatrics and adults with ASD [20, 74–76]. Interestingly, NRXN1 knockdown in primary cerebellar culture impairs synaptogenesis by altered interaction with glutamate receptor GluRdelta2 [77]. Mice NRXN1 knockdown models also show ASD-like abnormal social behaviors [78].

With respect to GM increase, we found a minor number of significant correlations between genes and RSNs compared to the decrease analysis. Despite the fact that this type of neuropathological phenomenon remains partially unclear in ASD and other psychiatric conditions [79], it is worth noting that the somatomotor, limbic, DMN and BG/Thal alterations present at least a significant correlation. These findings can be interpreted in light of previous functional connectivity studies, which report an abnormal over-connectivity in each one of these networks in autism [80, 81], again supporting a correspondence between the direction of connectivity changes and volumetric modifications.

With regard to the genes investigated, NLGN3 was the gene with the most overall significant correlations when considering both decrease and increase together. Interestingly, ASD cognitive deficits have been hypothesized to be correlated with altered NLGN family function [5, 6]. Specifically, NLGN3 encodes for a cell adhesion protein that is fundamental for proper synaptic transmission. The relation between this gene and ASD is supported by electrophysiological and molecular studies. For example, a study by Gutierrez and colleagues [82] found that a common ASD-mutation in NLGN3 decreases synchronicity between brain regions and affects axon architecture complexity, thereby decreasing neuronal connectivity.

NLGN4X, which we found to be significantly associated to DMN-related alteration in the decreases, and to the somatomotor-related RSN in the increases analyses, is known to be involved with ASD phenotypes by altering excitatory synaptic transmission due to its location in the postsynaptic density [13, 83]. However, its role in long-range connectivity and transmission of electric signaling is still not well understood, in part owing to the fact that NLGN4-like protein in mice co-localizes at inhibitory synapses, instead of excitatory as in humans [83]. Further insight into the role of this protein in the transmission of electric signaling would be beneficial in understanding the correlation with the areas of GM alteration found in the present study.

The neurexin superfamily (consisting in our data of NRXN1 and CNTNAP2, respectively) was also found to be significantly correlated with areas of altered GM in the DAN, DMN, Cerebellar, and the BG/Thal networks. CNTNAP2 is found mostly at the nodes of Ranvier and is involved with the electrochemical transmission in neurons [84]. Various CNTNAP2 polymorphisms have been found to predispose to ASD via altered long-range connectivity in the brain [84]. NRXN1 is a presynaptic protein which forms complexes with various post-synaptic proteins including the neuroligin family. NRXN1 heterozygous mutations have been found to reduce the brain metabolism and thus reduce the efficiency of connectivity in neural networks including thalamic, mesolimbic and cortical systems [85]. As previously mentioned, long-range disconnections between brain regions are often thought to be the etiology of neurodevelopmental disorder, including ASD [86–89].

We found that the SHANK family of scaffolding post-synaptic proteins to be significantly correlated to some canonical RSNs only in the decrease analysis. SHANK3 ASD-causative loss of functions mutations in particular has been found to be associated with low-functioning ASD, with both intellectual and language disability, on top of the social deficit [5, 6]. One of the underlying mechanisms may be the disruption of long- and short-range projections in the prefrontal and frontal striatal cortex areas associated with DMN [89]. This would reduce the connectivity in the prefrontal cortex, which Pagani and colleagues [90] linked to social communications deficits. Interestingly, a correspondence with our data can be noted: SHANK3 was found here to be significantly correlated with GM decrease clusters in the DMN, and Pagani and colleagues report that Shank3B^-/-^ mice have a decreased brain volume in DMN areas [90]. The role of SHANK1 in long-range connectivity is still not comprehensively understood, possibly because mutations in this gene may have less of an impact on synapse morphology, therefore causing a less severe phenotype than fellow SHANK family proteins [11]. Nonetheless, it is still known to alter post-synaptic protein composition, thereby reducing the size of the dendritic spines, weakening basal synaptic transmission [11, 91]. We did not find any significant correlations between SHANK2 and neuroanatomical data, even though it is known to be associated with ASD phenotypes [10]. This however, does not mean that it is not implicated with ASD. In fact, our results only imply that its expression in the altered GM regions is not associated to any specific network, that is, its effect might be homogeneous across RSNs.

Unlike the other genes considered, MECP2 encodes for a transcription factor. Autistic individuals with mutation in MECP2 have been found to have increased cerebellum volume [92]. It is hypothesized that loss of MECP2 leads to weakening of neuronal connections, therefore inhibiting spontaneous synaptic activity and weakening synaptic plasticity [93] which could explain the correlation found with areas of decreased GM.

Recently, Liloia and colleagues [94] have demonstrated in a meta-connectomic manner that GM abnormalities form a non-random network of co-alteration in individuals with ASD, which tends to overlap with the structural brain connectivity pathways. Interestingly, DMN was found to have a crucial topological role. Similarly, we found that the DMN was correlated with at least one candidate gene in increase and decrease analysis. It is also interesting to note that Liloia and colleagues [94] found no significant correlation between the GM co-alteration network and genetic co-expression connectivity. This may be because co-alteration is a different phenomenon than simple alteration. Another possible explanation is that the authors correlated one network to the entirety of the genome as found on AHBA, further supporting our rationale of selecting individual genes and testing them one by one.

### Methodological considerations and limitations

The current study proposes a new outlook on the brain pathobiology of ASD, extending previous research in several ways. The multimodal approach embraced here permits extending canonical information given by the ALE method, providing valuable insights on the mutual relationships between VBM abnormalities and genetic expressions of selected genes. In addition, shifting from a regional to large-scale network perspective, we have exerted a more comprehensive resolution of the autistic brain landscape, in line with the notion of ASD as a syndrome due to network-level disturbances [94–97]. Lastly, this work offers novel insights into brain architecture in ASD. Moreover, it is important to note that the current approach can be potentially applied to other disorders reporting appreciable structural brain atypicalities and genetic basis.

Despite these strengths, the present findings should be considered in the light of the following limitations. First, the meta-analytic and cross-sectional nature of VBM findings does not permit to evaluate the longitudinal sequence of GM variations across the lifespan in ASD and its link to gene expressions. At the same time, it should be noted that the CBMA approach tends to afford more robust and reliable results in terms of generalization for the clinical population of interest [98]. Second, although age-related and sex-related brain alterations have been previously noted in the disorder [74, 99], the design constraints of published VBM data hamper the possibility to perform subanalyses related to the age-stratified and sex-stratified populations. For instance, 32 out of 51 experiments (i.e. 63% of the total VBM dataset) analyzed both male and female subjects with ASD (S2 Table). Third, although potential bias regarding the VBM analysis of overlapping ASD populations was systematically minimized, we are unable to determine whether and to what extent a population overlap occurs, in particular for the efforts using subjects from the Autism Brain Imaging Data Exchange (ABIDE) database [100]. Fourth, correlational results are based exclusively on Yeo’s large-scale functional atlas and part of the Brainnettome’s atlas. Owing to random statistical fluctuation and possible different parcelings of neural populations, the results may change slightly when using a different atlas. Fifth, the genetic expression data obtained from the AHBA are characterized by a non-negligible degree of spatial uncertainty. Specifically, only two out of the six individuals have a bi-hemispheric mapping, and data were obtained with different stereotactic coordinates. To address these issues, we employed the Voronoi tassellation method and chose to average the genetic maps in a voxel-wise manner to reduce the variance among the gene expression patterns of the individuals, thus minimizing these weaknesses as much as possible. Sixth, genetic and VBM data analysed here did not come from the same cohort of subjects. However, Cauda et al. [29] had previously shown that comparing VBM meta-analytic data with functional and genetic data from different groups of subjects gives interpretable and statistically correct results. Finally, we only covered 8 out of the many putative ASD genes, with the intent of conducting an exploratory analysis on some of the genes that might be more representative of the disorder. Therefore, our findings are clearly not representative of the whole relation between ASD genetics and neuroanatomical variations. Future research may adopt the current pipeline to explore the relationship between a larger set of genes and brain networks, as well as to embrace a longitudinal design, to provide further insight into this complex disease.

## Conclusions

We conducted a coordinate-based meta-analysis using the ALE method to identify gray matter alterations in ASD. Having selected putative genes linked to ASD, we obtained typical gene expression patterns in healthy adults from the AHBA database. We correlated gene and structural alterations. The correlation coefficients of each voxel were projected in the Yeo’s cortical atlas and the mean coefficient for each resting-state network was calculated. We confirmed the role of DMN in the disorder, as it correlated with the gene expression of most genes both in areas with GM decrease and increase. Conversely, gene expression in areas in the DAN and cerebellar network correlated only with GM decrease, whereas in the somatomotor, ganglia/thalamus and limbic networks contained correlation between gene expression of a few genes and GM increase. This novel approach thus allowed us to combine structural and genetic information to study ASD and to identify meaningful correlations in functional networks.

## Supporting information

S1 Fig Map showing the gene expression of the selected genes. The maps are visualized as three-dimensional cortical and cerebellar surfaces. Brain templates are in neurological convention (i.e. R is right, L is left).
(DOCX)

S2 Fig PRISMA flow chart of meta-data selection. (DOCX)

S1 Table PRISMA checklist. (DOCX)

S2 Table Demographic characteristics of the studies included in the coordinate-based meta-analysis. (DOCX)

S3 Table Methodological characteristics of the studies included in the coordinate-based meta-analysis. (DOCX)

S4 Table Diagnostic labeling of the studies included in the coordinate-based meta-analysis. (DOCX)
