# Supplementary material for: Linking neuroanatomical abnormalities in autism spectrum disorder with gene expression of candidate ASD genes: A meta-analytic and network-oriented approach
Source: PLoS One. 2022 Nov 28;17(11):e0277466. doi: 10.1371/journal.pone.0277466 (PMC9704678; doi:10.1371/journal.pone.0277466)
Supplement: S1 Fig — The maps are visualized as three-dimensional cortical and cerebellar surfaces. Brain templates are in neurological convention (i.e. R is right, L is left). (DOCX) [file pone.0277466.s001.docx]

**
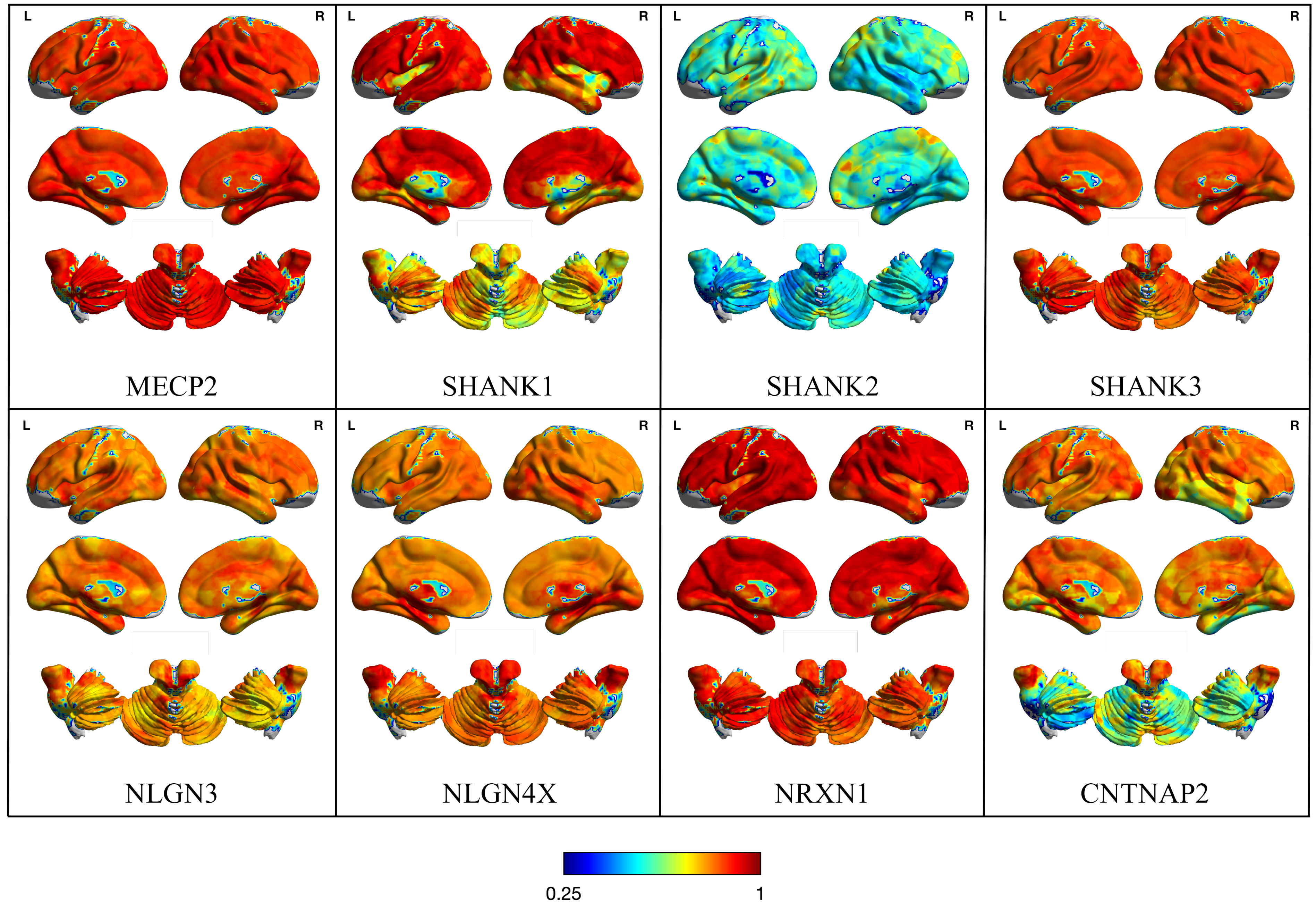
**

**Figure S1.** Map showing the gene expression of the selected genes. The maps are visualized as three-dimensional cortical and cerebellar surfaces. Brain templates are in neurological convention (i.e. R is right, L is left).
