# Supplementary material for: Linking neuroanatomical abnormalities in autism spectrum disorder with gene expression of candidate ASD genes: A meta-analytic and network-oriented approach
Source: PLoS One. 2022 Nov 28;17(11):e0277466. doi: 10.1371/journal.pone.0277466 (PMC9704678; doi:10.1371/journal.pone.0277466)
Supplement: S2 Fig — (DOCX) [file pone.0277466.s002.docx]

**BrainMap.org VBM Database**

(Sleuth systematic search)

Query A (ASD < TDCs)

Query B (ASD > TDCs)

**MEDLINE Database**

(PubMed advanced search)

Potentially relevant articles
(n = 517)

Title and abstract screening
(n = 427)

Full-text articles screening
(n = 209)

Articles excluded:

Not VBM analysis (n= 165)

Only white matter analysis (n= 25)

Non-human (n= 24)

Full-text not available (n= 4)

Articles excluded:
Duplicates (n=90)

Articles excluded:

Fewer than 10 subjects (n=2)

Sample overlap (n=2)

Foci not reported (n=3)

ROI analysis (n=6)

Only white matter analysis (n= 14)

Not VBM analysis (n=134)

51 Articles included*

For a total of:

° GM decreases experiments (n=45)

° GM increases experiments (n=34)

*25 articles reported two independent ASD groups

Articles identified via reviews and meta-analyses (n= 3)

**Figure S2.** PRISMA flow chart of meta-data selection.

VBM: voxel-based morphometry; ASD: autism spectrum disorder; TDCs: typically developing controls; GM: gray matter; ROI: region-of-interest.
