# Supplementary material for: Linking neuroanatomical abnormalities in autism spectrum disorder with gene expression of candidate ASD genes: A meta-analytic and network-oriented approach
Source: PLoS One. 2022 Nov 28;17(11):e0277466. doi: 10.1371/journal.pone.0277466 (PMC9704678; doi:10.1371/journal.pone.0277466)
Supplement: S2 Table — (DOCX) [file pone.0277466.s004.docx]

**Table S2.** Demographic characteristics of the studies included in the coordinate-based meta-analysis.

| **ID**  **Study** | **First**  **Author** | **ASD Sample** | | | | **Healthy Sample** | | | |
| --- | --- | --- | --- | --- | --- | --- | --- | --- | --- |
|  |  | **N** | **Age at scan**  **(years)** † | **M** | **F** | **N** | **Age at scan**  **(years)** † | **M** | **F** |
| 1 | Abell F | 15 | 29±6.6 | 12 | 3 | 15 | 25±3.1 | 12 | 3 |
| 2 | Boddaert N | 21 | 9.3±2.2 | 16 | 5 | 12 | 10.8±2.7 | 7 | 5 |
| 3 | Bonilha L | 12 | 12.4±4 | 12 | 0 | 16 | 13.2±5 | 11 | 5 |
| 4 | Brieber S | 15 | 14.2±1.9 | 15 | 0 | 15 | 13.3 | 15 | 0 |
| 5 | Cai J | 38 | 9.6±3.4 | 32 | 6 | 27 | 8.3±2.3 | 26 | 1 |
| 6 | Calderoni S | 38 | 4.4±1.5 | 0 | 38 | 38 | 4.4±1.4 | 0 | 38 |
| 7 | Cheng Y | 25 | 13.7±2.5 | 25 | 0 | 25 | 13.5±2.1 | 25 | 0 |
| 8 | Craig M C | 14 | 37.9±11.4 | 0 | 14 | 19 | 35±14 | 0 | 19 |
| 9 | D'Mello A M | 35 | 10.4±1.6 | 30 | 5 | 35 | 10.4±1.5 | 21 | 14 |
| 10 | D'Mello A M | 18 | 11.0±1.6 | 18 | 0 | 35 | 10.4±1.5 | 21 | 14 |
| 11 | Ecker C | 22 | 27±7 | 22 | 0 | 22 | 28±7 | 22 | 0 |
| 12 | Ecker C | 89 | 26±7 | 89 | 0 | 89 | 28±7 | 89 | 0 |
| 13 | Eilam-Stock T | 66 | 27±8 | 60 | 6 | 66 | 27±7 | 60 | 6 |
| 14 | Foster N E | 38 | 12.4±2.4 | 38 | 0 | 46 | 12.6±2.6 | 46 | 0 |
| 15 | Freitag C M | 15 | 17.6±3.6 | 13 | 2 | 15 | 18.6±1.2 | 13 | 2 |
| 16 | Greimel E | 47 | 21.4±10.1 | 47 | 0 | 51 | 18.3±7.5 | 51 | 0 |
| 17 | Hyde K L | 15 | 22.7±6.4 | 15 | 0 | 13 | 19.2±5 | 13 | 0 |
| 18 | Katz J | 23 | 26.6±6.5 | 23 | 0 | 32 | 29.8±9.2 | 32 | 0 |
| 19 | Kaufmann L | 10 | 14.7±5.0 | 8 | 2 | 10 | 13.8±5.3 | 8 | 2 |
| 20 | Ke X | 17 | 8.88±1.96 | 14 | 3 | 15 | 9.73±1.67 | 12 | 3 |
| 21 | Kosaka H | 32 | 23.8±4.2 | 32 | 0 | 40 | 22.5±4.3 | 40 | 0 |
| 22 | Kurth F | 52 | 11.2±3.95 | 38 | 14 | 52 | 11.14±3.58 | 38 | 14 |
| 23 | Kwon H | 11 | 13.6±2.4 | 11 | 0 | 13 | 13.6±3.1 | 13 | 0 |
| 24 | Lai M C | 60 | 27.5 | 30 | 30 | 60 | 27.8 | 30 | 30 |
| 25 | Lai M C | 80 | 24.2 | 80 | 0 | 57 | - | 57 | 0 |
| 26 | Lim L | 19 | 14.9±1.8 | 19 | 0 | 33 | 14.3±2.5 | 33 | 0 |
| 27 | Lin H Y | 18 | 13.3±2.5 | 18 | 0 | 48 | 12.8±2.6 | 54 | 0 |
| 28 | McAlonan G M | 21 | 32±10 | 19 | 2 | 24 | 33±7 | 22 | 2 |
| 29 | McAlonan G M | 17 | 12±1.8 | 16 | 1 | 17 | 11±1.2 | 16 | 1 |
| 30 (exp. A) | McAlonan G M | 17 | 11.4±2.5 | 14 | 3 | 55 | 10.7±2.7 | 47 | 8 |
| 30 (exp. B) | McAlonan G M | 16 | 11.7±2.8 | 13 | 3 |  |  |  |  |
| 31 | Mengotti P | 20 | 7.0±2.7 | 18 | 2 | 22 | 7.7±2.0 | 20 | 2 |
| 32 | Mueller S | 12 | 35.5±11.4 | 9 | 3 | 12 | 33.3±9 | 8 | 4 |
| 33 | Ni H C | 81 | 12.5±2.1 | 81 | 0 | 61 | 12.4±2.4 | 61 | 0 |
| 34 | Osipowicz K | 531 | 17.0±8.0 | 430 | 101 | 571 | 17.0±8.0 | 462 | 109 |
| 35 | Pappaianni E | 39 | 10.0±1.0 | 39 | 0 | 42 | 10.0±1.0 | 42 | 0 |
| 36 | Pereira A M | 22 | 17.4±3.3 | 18 | 4 | 29 | 18.5± 2.8 | 19 | 10 |
| 37 | Radeloff D | 34 | 19.06±5.12 | 31 | 3 | 26 | 19.54±3.46 | 22 | 4 |
| 38 | Riddle K | 390 | 18.6±8.7 | 341 | 69 | 443 | 18.3±7.8 | 362 | 81 |
| 39 | Riedel A | 30 | 35.4±9.1 | 19 | 11 | 30 | 35.5±8.3 | 19 | 11 |
| 40 | Riva D | 21 | 6.6±2.5 | 13 | 8 | 21 | 6.10±2.1 | 13 | 8 |
| 41 | Riva D | 26 | 5.1±2.6 | 23 | 3 | 21 | 6.10±2.1 | 13 | 8 |
| 42 | Rojas D C | 24 | 20.79±10.58 | 24 | 0 | 23 | 21.41±10.91 | 23 | 0 |
| 43 | Salmond C H | 14 | 12.9±0.7 | 13 | 1 | 13 | 12.1±0.7 | 13 | 0 |
| 44 | Salmond C H | 22 | 11.8 | 20 | 2 | 22 | 12.1 | 19 | 3 |
| 45 | Sato W | 36 | 27.0±8.0 | 25 | 11 | 36 | 24.9±5.5 | 25 | 11 |
| 46 | Schmitz N | 10 | 38±9 | 10 | 0 | 10 | 39±6 | 10 | 0 |
| 47 (exp. A) | Toal F | 39 | 32.0±12.0 | 35 | 4 | 33 | 30.0±3.0 | 30 | 3 |
| 47 (exp. B) | Toal F | 26 | 30.0±8.0 | 21 | 5 |  |  |  |  |
| 48 | Waiter G D | 16 | 15.4±2.24 | 16 | 0 | 16 | 15.5±1.6 | 16 | 0 |
| 49 | Wang J | 31 | 4.8±1.1 | 31 | 0 | 31 | 4.8± 0.8 | 31 | 0 |
| 50 | Wilson L B | 10 | 30.1±9.18 | 8 | 2 | 10 | 29.4±7.91 | 7 | 3 |
| 51 | Yang Q | 16 | 10.4± 2.8 | 10 | 6 | 16 | 10.5 ± 3.1 | 10 | 6 |
| **Total** | | **2366** |  | **2014** | **372** | **2483** |  | **2059** | **430** |

† The average age and its standard deviation or the age range are reported on the basis of what is specified by the authors. N = number of subjects; M = male; F = female.
