# Supplementary material for: Linking neuroanatomical abnormalities in autism spectrum disorder with gene expression of candidate ASD genes: A meta-analytic and network-oriented approach
Source: PLoS One. 2022 Nov 28;17(11):e0277466. doi: 10.1371/journal.pone.0277466 (PMC9704678; doi:10.1371/journal.pone.0277466)
Supplement: S3 Table — (DOCX) [file pone.0277466.s005.docx]

**Table S3.** Methodological characteristics of the studies included in the coordinate-based meta-analysis.

| **Article**  **ID** | **First**  **Author** | **VBM**  **Software** | **Voxel Size**  **(mm)** † | **Smoothing**  **FWHM** | **Scanner**  **(Tesla)** | **Original**  **Coordinates** |
| --- | --- | --- | --- | --- | --- | --- |
| 1 | Abell F | SPM96 | 1x1x1.5 | 12 | 2 | TAL |
| 2 | Boddaert N | SPM99 | 0.86x1.33x1.20 | 12 | 1.5 | TAL |
| 3 | Bonilha L | SPM5 | 1x1x1 | 8 | 2 | MNI |
| 4 | Brieber S | SPM2 | 1x1.42x1 | 12 | 1.5 | MNI |
| 5 | Caj J | SPM 8 | 1x1x1 | 8 | 3 | MNI |
| 6 | Calderoni S | SPM 8 | Thickness 1 | 8 | 1.5 | MNI |
| 7 | Cheng Y | SPM 2 | Thickness 1.5 | 8 | 1.5 | MNI |
| 8 | Craig M C | SPM2 | Thickness 1.5 | 5 | 1.5 | TAL |
| 9 | D'Mello A M | SPM 8 | 1x1x1 | 8 | 3 | MNI |
| 10 | D'Mello A M | SPM 8 | 1x1x1 | 8 | 3 | MNI |
| 11 | Ecker C | SPM5 | 1.09x1.09x1 | 8 | 3 | TAL |
| 12 | Ecker C | FSL-VBM | Thickness 1 | 3 | 3 | MNI |
| 13 | Eilam-Stock T | SPM 8 | - | 8 | - | MNI |
| 14 | Foster N E | CIVET | 1x1x1 | 8 | 3 | MNI |
| 15 | Freitag C M | SPM 99 | 1x1x1 | 8 | 1.5 | MNI |
| 16 | Greimel E | SPM5 | 1x1x1 | 8 | 1.5/3 | MNI |
| 17 | Hyde K L | CIVET | 1x1x1 | 12 | 3 | MNI |
| 18 | Katz J | FS-VBM | 1x1x1 | 7 | 3 | MNI |
| 19 | Kaufmann L | SPM 8 | - | 6 | 1.5 | MNI |
| 20 | Ke X | SPM5 | 0.94x0.94x1 | 8 | 1.5 | TAL |
| 21 | Kosaka H | SPM5 | 0.75x1.25x1.06 | 8 | 3 | MNI |
| 22 | Kurth F | SPM 8 | Thickness 1.2 | 8 | 1.5 | MNI |
| 23 | Kwon H | SPM99 | Thickness 1.5 | 8 | 3 | TAL |
| 24 | Lai M C | SPM 8 | - | 4 | 3 | MNI |
| 25 | Lai M C | SPM 8 | 1x1x1 | 4 | 3 | MNI |
| 26 | Lim L | SPM 8 | - | 8 | 3 | MNI |
| 27 | Lin H Y | SPM 8 | Thickness 3 | 4 | 3 | MNI |
| 28 | McAlonan G M | SMaRT on SPARC | Thickness 1.5 | - | 1.5 | TAL |
| 29 | McAlonan G M | BAMM on SPARC | Thickness 3 | 4.4 | 1.5 | TAL |
| 30 | McAlonan G M | BAMM on SPARC | 0.86x0.86x3 | 4.4 | 1.5 | TAL |
| 31 | Mengotti P | SPM 5 | Thickness 5 | 8 | 1.5 | MNI |
| 32 | Mueller S | FSL-VBM | 0.8x0.8x0.8 | 4 | 3 | MNI |
| 33 | Ni H C | SPM 8 | 1x1x1 | 4 | 3 | MNI |
| 34 | Osipowicz K | SPM 8 | 1x1x1 | 8 | 3 | MNI |
| 35 | Pappaianni E | SPM 12 | - | 8 | - | MNI |
| 36 | Pereira A M | SPM 8 | 1x1x1 | 10 | 3 | MNI |
| 37 | Radeloff D | SPM 8 | Thickness 1 | 8 | 3 | MNI |
| 38 | Riddle K | SPM 8 | - | - | - | MNI |
| 39 | Riedel A | SPM 8 | 1x1x1 | 8 | - | MNI |
| 40 | Riva D | SPM 5 | 1x1x1 | 8 | 1.5 | TAL |
| 41 | Riva D | SPM 8 | Thickness 5 | 8 | 1.5 | TAL |
| 42 | Rojas D C | SPM2 | 0.94x0.94x1.7 | 8 | 1.5 | MNI |
| 43 | Salmond C H | SPM99 | 0.8x0.8x1 | 12 | 1.5 | TAL |
| 44 | Salmond C H | SPM99 | 0.8x0.8x1 | 12 | 1.5 | MNI |
| 45 | Sato W | SPM 8 | 1x1x1 | 8 | 3 | MNI |
| 46 | Schmitz N | SPM 99 | 2x2x2 | 10 | 1.5 | TAL |
| 47 | Toal F | SPM 2 | 0.859x0.859x1.5 | 8 | 1.5 | TAL |
| 48 | Waiter G D | SPM2 | 1x1x1 | 12 | 1.5 | TAL |
| 49 | Wang J | SPM 12 | 1x1x1 | 6 | 3 | MNI |
| 50 | Wilson L B | SPM2 | 0.94x0.94x1.7 | 12 | 1.5 | MNI |
| 51 | Yang Q | FS-VBM | Thickness 1 | 3 | 3 | MNI |

† Where no information was provided, the slice thickness was expressed. FWHM = full width at half maximum; TAL = Talairach stereotactic space; MNI = Montreal Neurological Institute stereotactic space.
