# Supplementary material for: Linking neuroanatomical abnormalities in autism spectrum disorder with gene expression of candidate ASD genes: A meta-analytic and network-oriented approach
Source: PLoS One. 2022 Nov 28;17(11):e0277466. doi: 10.1371/journal.pone.0277466 (PMC9704678; doi:10.1371/journal.pone.0277466)
Supplement: S4 Table — (DOCX) [file pone.0277466.s006.docx]

**Table S4.** Diagnostic labeling of the studies included in the coordinate-based meta-analysis.

| **Article ID** | **First Author** | **Diagnosis†** | **ASD < HC** | **ASD > HC** |
| --- | --- | --- | --- | --- |
| 1 | Abell F | ASP | 3 | 6 |
| 2 | Boddaert N | Autism | 4 | 0 |
| 3 | Bonilha L | ASD | 0 | 69 |
| 4 | Brieber S | ASP/HFA | 6 | 2 |
| 5 | Cai J | HFA | 3 | 3 |
| 6 | Calderoni S | Autism/PDD-NOS | 0 | 1 |
| 7 (exp. A) | Cheng Y | HFA | 2 | 2 |
| 7 (exp. B) | Cheng Y | ASP | 10 | 11 |
| 8 | Craig M C | ASP/Autism | 5 | 0 |
| 9 | D'Mello A M | ASD | 3 | 3 |
| 10 | D'Mello A M | ASD | 2 | 0 |
| 11 | Ecker C | ASD | 14 | 11 |
| 12 | Ecker C | ASP/HFA | 11 | 26 |
| 13 | Eilam-Stock T | HFA | 3 | 10 |
| 14 | Foster N E | ASD | 5 | 41 |
| 15 | Freitag C M | ASD | 1 | 0 |
| 16 | Greimel E | ASP/HFA /Autism | 3 | 0 |
| 17 | Hyde K L | Autism | 3 | 8 |
| 18 | Katz J | HFA | 0 | 2 |
| 19 | Kaufmann L | ASP | 1 | 2 |
| 20 | Ke X | HFA | 1 | 6 |
| 21 | Kosaka H | ASP/HFA | 5 | 0 |
| 22 | Kurth F | ASD | 1 | 0 |
| 23 | Kwon H |  | 3 | 0 |
| 24 | Lai M C | ASP/Autism | 1 | 1 |
| 25 | Lai M C | ASP/Autism | 6 | 7 |
| 26 | Lim L | ASD | 0 | 2 |
| 27 | Lin H Y | ASD | 0 | 1 |
| 28 | McAlonan G M | ASP | 9 | 0 |
| 29 | McAlonan G M | Autism | 13 | 0 |
| 30 (exp. A) | McAlonan G M | HFA | 8 | 0 |
| 30 (exp. B) | McAlonan G M | ASP | 4 | 0 |
| 31 | Mengotti P | Autism | 2 | 7 |
| 32 | Mueller S | HFA | 14 | 0 |
| 33 | Ni H C | HFA | 6 | 3 |
| 34 | Osipowicz K | ASD | 5 | 12 |
| 35 | Pappaianni E | ASD | 0 | 6 |
| 36 | Pereira A M | HFA | 16 | 2 |
| 37 | Radeloff D | ASP/Autism | 4 | 1 |
| 38 | Riddle K | ASD | 0 | 1 |
| 39 | Riedel A | HFA | 1 | 0 |
| 40 | Riva D | Autism/PDD-NOS | 13 | 0 |
| 41 | Riva D | Autism/PDD-NOS | 13 | 0 |
| 42 | Rojas D C | 24 AUTISM | 4 | 9 |
| 43 | Salmond C H | ASP/HFA | 2 | 16 |
| 44 | Salmond C H | ASD | 6 | 4 |
| 45 | Sato W | ASP/PDD-NOS | 19 | 0 |
| 46 | Schmitz N | ASP/HFA | 0 | 5 |
| 47 (exp. A) | Toal F | ASP | 1 | 0 |
| 47 (exp. B) | Toal F | ASD | 2 | 2 |
| 48 | Waiter G D | ASP | 1 | 13 |
| 49 | Wang J | ASD | 0 | 2 |
| 50 | Wilson L B | Autism | 2 | 0 |
| 51 | Yang Q | ASD | 3 | 0 |
| **Total** | | | **244** | **297** |

† Reported diagnoses are based on what is specified by the authors. ASD < HC = foci of GM decrease; ASD > HC = foci of GM increase. ASP = Asperger’s Syndrome; AUTISM = Primary Autism; HFA = High Functional Autism; ATYPIC AUTISM = Atypical Autism; ASD = Autism Spectrum Disorder (diagnosis not specified); PDD-NOS = Pervasive Developmental Disorder Not Otherwise Specified.
